# New Insights Into Physiological and Pathophysiological Functions of Stanniocalcin 2

**DOI:** 10.3389/fendo.2020.00172

**Published:** 2020-03-31

**Authors:** Aditya D. Joshi

**Affiliations:** Department of Pharmacology and Toxicology, University of Texas Medical Branch, Galveston, TX, United States

**Keywords:** stanniocalcin 2, calcium regulation, development, angiogenesis, cytoprotection, apoptosis, tumor biology

## Abstract

Stanniocalcin, a glycosylated peptide hormone, first discovered in a bony fish has originally been shown to play critical role in calcium and phosphate homeostasis. Two paralogs of stanniocalcin (*STC1* and *STC2*) identified in mammals are widely expressed in variety of tissues. This review provides historical perspective on the discovery of fish and mammalian stanniocalcin, describes molecular regulation of *STC2* gene, catalogs distribution as well as expression of STC2 in tissues, and provides key structural information known till date regarding mammalian STC2. Additionally, this mini review summarizes pivotal functions of STC2 in calcium and phosphate regulation, cytoprotection, cell development, and angiogenesis. Finally, STC2's role as a novel marker for human cancers has also been outlined. Reviewing these studies will provide an opportunity to understand STC2's structure, biological functions as well as key molecular pathways involving STC2, which will help us design innovative therapeutic interventions using this novel hormone.

## Introduction

The stanniocalcin—historically known as hypocalcin, teleocalcin, or parathyrin—is a widely-expressed hormone that is speculated to function in an autocrine and/or paracrine manner (1, 2). Various studies have indicated the possible involvement of two mammalian stanniocalcins, namely stanniocalcin 1 and 2 (STC1 and 2), in diverse biological processes including calcium regulation, cell proliferation and apoptosis, inflammation, Endoplasmic Reticulum (ER)/oxidative stress, metabolism, and cancer (3–10). However, precise physiological functions and signaling pathways in which stanniocalcins are involved remain to be elucidated. Majority of previous studies and reports were centered on understanding role of STC1 in plethora of cellular and molecular functions (11–17). This mini review is entirely focused on depicting our current understanding of physiological and pathological role of STC2 within human health and disease context.

## Discovery Of Mammalian Stanniocalcins

Stanniocalcin is a glycosylated, disulfide-linked, homodimeric hormone, first isolated from the corpus of stannous—a small endocrine gland in the kidney of teleostean and holostean fish (1, 2, 18–21). In 1964, Fontaine et al., “stannioectomized” (surgically removed) corpus of stannous and observed hypercalcemia along with decrease in sodium and chloride levels (22). Further studies identified stanniocalcin as a bonafide regulator of calcium entry through gills and intestine (23, 24). It was also shown that stanniocalcin mediated decrease in cAMP resulted in inhibition of calcium channels at gill epithelial cells (25). In 1986, Wagner et al. and Lafeber et al. successfully purified and characterized stanniocalcin from corpus of stannous of salmon and trout respectively (2, 20). However, no corpus of stannous or similar glands were identified in mammals and therefore it was assumed that stanniocalcin gene was lost during evolution. In 1996, Yoshiko et al. observed that the accumulation of cAMP induced by parathyroid hormone in ROS 17/2.8-5 cells was suppressed by N-terminal synthetic stanniocalcin from *Oncorhynchus keta* (chum salmon) (26). These data for the first time demonstrated that the fish hormone, stanniocalcin has a biological activity in isolated rat cells and its biological function of calcium regulation is intact. The presence of mammalian stanniocalcin was confirmed by Reddel laboratory when first human and later mouse stanniocalcin cDNA were cloned (27, 28). The amino acid sequence of mammalian stanniocalcin is 61% homologous to that of fish stanniocalcin. In 1998, stanniocalcin 2 (*STC2*), a paralog of stanniocalcin (later renamed as *STC1*) was identified by two independent groups (4, 29, 30). Ishibashi et al. cloned *STC2* from human osteosarcoma cDNA library, whereas Reddel research group identified, cloned and characterized *STC2* from both human and mouse. Human STC2 showed 34% identity with human STC1 as well as with eel stanniocalcin (4).

## Molecular Regulation of *STC2* Gene

Human *STC2* gene is located on chromosome 5q35.1, whereas *STC1* is located on chromosome 8p21.2 (31). Both human and mouse *STC2* contains 4 exons spanning 13 kb of DNA. It was observed that the exon-intron boundaries, distribution of cysteine residues and the glycosylation site were conserved between *STC1* and 2 and subsequent genomic structure analysis indicated that both paralogs were derived from common ancestor gene (32). *STC2* lacks the well-defined CAG repeats as well as the TATA box-like sequences present in the *STC1* (4). Very few studies have interrogated genetic regulation of *STC2*. DiMattia's laboratory identified that in human breast carcinoma cell lines T-47D and MCF7—estrogen (E2), progesterone (P4), and retinoic acid (RA) receptors play critical role in the regulation of *STC2* (33). Promoter analysis revealed absence of estrogen, progesterone, or RA receptor elements in the proximal promoter region of the *STC2* gene indicating that regulation of *STC2* by these upstream receptors is a secondary response (33). Chromatin immunoprecipitation studies indicated binding of Hypoxia inducible factor 1α (HIF1α) to the *STC2* promoter which contains Hypoxia Response Elements (HRE) (7). This study confirmed *STC2* as a HIF1α target gene that promotes cell proliferation in hypoxia in human breast and ovarian cancer cells (34). HIF1α induced STC2 expression is modulated by two cofactors namely, histone acetyltransferase p300 and histone deacetylase 7 (HDAC7) (34). Additionally, our laboratory showed *Stc2* as an Aryl hydrocarbon Receptor (AhR) target gene containing Xenobiotic Response Elements (XRE) (35). *Stc2* promoter contains 8 XREs clustered in a 250-bp region that was shown to recruit AhR by chromatin immunoprecipitation (35). Lastly, a study performed in mice deficient in klotho showed that *klotho* gene expression played important role in the regulation of renal *Stc2* gene through the control of calcium and phosphorous concentrations (36).

## Structure, Expression, and Distribution of STC2

**Figure 1:**
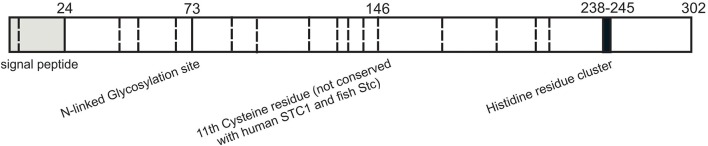
Structural features of stanniocalcin 2. Schematic representation of the known functional residues of human STC2. Putative signal peptide sequence is shown in gray. N-Glycosylation site is denoted with solid line and 15 cysteine residues are represented with dashed lines. Cluster of histidine residues (HHxxxxHH) is in black.

Human and mouse STC2 proteins are 302 and 296 amino acids in length respectively with first 24 residues predicted to be a signal peptide and remaining residues comprise the mature form of the hormone. STC2, a 56kDa protein has no sequence homology with parathyroid hormone (25). The hallmarks of STC2 are the cysteine residues conserved among family members and N-linked glycosylation consensus sequence (Asn-X-Thr/Ser) (Figure 1) (37). STC2 have 15 cysteines, whereas STC1 and fish stanniocalcin have 11 cysteines. The locations of first 10 cysteines are conserved within the stanniocalcin family. However, the 11th cysteine residue conserved between STC1 and fish stanniocalcin is not spatially conserved in STC2 (18). This cysteine plays crucial role in disulfide-linked homodimer formation (18). Therefore, it is predicted that the tertiary structure of STC2 might be different than that of STC1 and fish stanniocalcin. So far, no studies have shown a heterodimer formation between STC1 and STC2. STC2 is also phosphorylated by casein kinase 2 on its serine residues (38). The C-terminal of STC2 has a cluster of histidine residues (HHxxxxHH), which may interact with divalent metal ions such as cobalt, copper, nickel, and zinc—though the functional significance of this cluster remained to be studied (37). In mammals, investigation of tissue distribution of *STC2* mRNA revealed its expression in variety of tissues including pancreas, heart, placenta, spleen, lung, kidneys, and skeletal muscles (4, 30, 39). Additionally, abundant STC2 protein expression was observed in brain, lungs, liver, and kidneys (4, 30). An immunohistochemical study indicated STC2 expression and co-localization with glucagon-secreting alpha cells in pancreatic islets, strongly indicating STC2's involvement in glucose homeostasis (37). Additionally, various tumor cell lines specifically from lungs, colon, and mammary glands reported upregulated levels of STC2 (40–43).

Several reports suggest that STC2 is a secreted protein based on the findings with its paralog, STC1 (12). Human fibrosarcoma cell line, HT1090 has shown to secrete both STC1 and STC2 as phosphoproteins in the medium (38). Initial subcellular fractionation and immunogold labeling studies indicated localization of stanniocalcin to the inner mitochondrial matrix (12). In transfected COS cells, STC2 localization to the ER and Golgi apparatus was demonstrated—consistent with its secretary fate (5). This report however failed to overlap STC2 with mitochondrial markers in COS cells. Purified mitochondrial fractions were also devoid of any STC2 expression (5). Immunofluorescence studies performed on permeabilized mouse primary hepatocytes treated with cinnabarinic acid, an Aryl hydrocarbon Receptor agonist, to induce STC2 expression revealed presence of STC2 prominently in ER based on co-localization with ER marker, KDEL (6). On contrary, non-permeabilized hepatocytes displayed punctate staining of STC2 on cell surface. Confocal microscopy performed on liver sections also showed STC2 puncta associated with the plasma membrane based on the overlapping distribution with membrane marker pan-cadherin (6). Furthermore, a stanniocalcin-alkaline phosphatase (STC-AP) fusion protein was generated to identify and localize stanniocalcin receptor (12). In mouse outer medullary kidney sections, binding of STC-AP to thick ascending limb cells and collecting duct cells were observed. Mouse outer cortical kidneys showed specific punctate binding over distal convoluted tubules and collecting duct cells. Apical membrane staining was also noticed in proximal convoluted tubules. In liver, putative stanniocalcin receptor was present in hepatocytes as detected by punctate staining, especially on cell membranes near central vein (12). Therefore, based on the current immunological data, it is conceivable that STC2 is bound to the putative stanniocalcin receptor(s) and might function in an autocrine and/or paracrine fashion as speculated by various groups (5, 6, 12, 44).

## Physiological and Pathological Functions of STC2

### Phosphate and Calcium Regulation

Studies performed in cell lines have provided the evidence of STC2's involvement in kidney phosphate regulation. *STC2* transfected CHO cells inhibited the promoter activity of type II sodium phosphate transporters, present on the apical membranes of kidney proximal tubules (4). Inhibition of type II sodium phosphate transporters resulted in reduction of phosphate uptake in opossum kidney cell lines (4). Therefore, current data suggests that STC2 inhibits phosphate transport through transcriptional regulation of phosphate transporter. Additionally, *Stc2* expression was downregulated in mice with hypophosphatemia (Phex^Hyp^), whereas mice on high-phosphate diet resulted in increased kidney *Stc2* mRNA expression (4). These studies strongly indicate that STC2 may play critical role in phosphate metabolism. Additionally, *STC2* overexpression in MC3T3 cells facilitated osteoblast differentiation and mineralization by regulation of ERK phosphorylation, suggesting STC2's involvement in bone metabolism (45). Experiments performed in mouse embryonic fibroblasts cultured from *Stc2* knockout animals displayed elevated levels of cytosolic calcium following ER calcium store depletion attributed to the increase in extracellular calcium influx through store operated calcium channels (9). Mouse embryonic fibroblasts that overexpress *Stc2* attenuated store operated calcium entry. Zeiger et al. further showed that STC2 interacted with Stromal interaction molecule 1 (STIM1), an ER calcium sensor, which triggered store operated calcium channels following ER store depletion (9). STC2 interaction with STIM1-ORAI (encoded by Orai3, Calcium Release-Activated Calcium Modulator 3) and subsequent store operated calcium entry was also observed in mouse platelets, where STC2 altered non-capacitative calcium entry and platelet aggregation by modulating expression of ORAI3 channels (46).

### Role in Animal Development

Both STC1 and 2 have been implicated in the regulation of tissue remodeling in mice (47–50). STC2 was specifically shown to attenuate ovarian progesterone biosynthesis via PKA pathway (51). The STC2 suppression of progesterone production was associated with the inhibition of follicle-stimulating hormone (FSH)-induced *Cyp1a1* and 3β-hydroxysteroid dehydrogenase expression (51). STC2 also interacted with Ran-binding protein M (RanBPM) and stimulated RanBPM-induced androgen receptor activation (52). Phenotypically *STC2* overexpressed mice exhibit growth restriction, whereas knockout mice were larger than wild-type littermates (53, 54). *Stc2* knockout mice were 10–15% larger and grew at a faster rate than its wild-type counterparts from four week onwards (53). On contrary, human *STC2* transgenic mice were 45% smaller than wild-type littermates (54). STC2's negative regulation of postnatal growth was demonstrated by its ability to interact with pregnancy-associated plasma protein-A (PAPP-A), potentially inhibiting its proteolytic activity toward insulin-like growth factor binding protein 4 (IGFBP4) and causing reduction in insulin-like growth factor (IGF) signaling (55, 56). Recent genome-wide association studies identified rare height increasing alleles of *STC2* with compromised proteolytic inhibition of PAPP-A and increased cleavage of IGFBP4 resulting in higher bioavailability of IGF (57). Additionally, STC2 mediated PAPP-A inhibition was also demonstrated to ameliorate atherosclerosis in hypercholesterolemic mice (58).

### Angiogenesis

Both STC1 and 2 showed stimulatory effects on angiogenesis (59). *STC2* overexpression in human umbilical vascular endothelial cells showed significant increase in cell cycle regulators—cyclin-D, phospho-retinoblastoma, matrix metalloproteinase 2 (MMP2), and decrease in tissue inhibitors of metalloproteases 1 (TIMP1). Furthermore, STC2 mediated angiogenic sprouting was due to activation of both Vascular endothelial growth factor/Vascular endothelial growth factor C (VEFG/VEGF2) and angiopoietin 2 pathways (59).

### Involvement in Cytoprotection

First evidence of STC2's cytoprotective function was demonstrated by Thinakaran et al. (5), where *STC2* was upregulated in N2a mouse neuroblastoma cell lines upon exposure to tunicamycin and thapsigargin induced ER stress. *STC2* was also elevated in response to H_2_O_2_ induced oxidative stress and hypoxia (5). Both in N2a and HeLa cell lines overexpression of STC2 protected cells from thapsigargin-induced cell death (5). Human neural crest derived Paju cells transfected with *STC2* showed increased resistance to ischemic challenge and thapsigargin induced stress (60). In mouse primary hepatocytes, activation of Aryl hydrocarbon Receptor by the tryptophan catabolite, cinnabarinic acid upregulated expression of STC2 to elicit cytoprotection against apoptosis induced by H_2_O_2_, thapsigargin and ethanol (6). Cinnabarinic acid treatment also provided protection against apoptosis and liver injury in mouse model of acute alcohol-induced hepatotoxicity. This *in vivo* cinnabarinic acid mediated cytoprotection was shown to be AhR-dependent and STC2 mediated albeit the exact pro-survival pathways downstream of STC2 need to be studied (6, 61). In a cerulean-induced pancreatitis mouse model, STC2 elevation was observed within four hours of initiating pancreatic injury (3). Elevated STC2 altered both protein kinase R (PKR)-like endoplasmic reticulum kinase (PERK) phosphorylation and activating transcription factor 4 (ATF4) levels though an undetermined mechanism and reduced acinar cell damage during pancreatic injury (3). In human adipose-derived mesenchymal stem cells (ADSC) and human mesenchymal stem cells isolated from umbilical cord blood (UCB-MSC) subjected to H_2_O_2_ induced oxidative stress, STC2 overexpression exhibited increased cell viability and survival. Upregulation of Cyclin-dependent kinase 2 and 4 (CDK2 and 4) as well as down-regulation of cell cycle inhibitors p16 and p21 were observed after *STC2* transduction. STC2 overexpression also resulted in activation of pAKT and pERK1/2 signaling pathways to protect against oxidative stress induced apoptosis (62). Our group also observed that cinnabarinic acid mediated upregulation of STC2 can protect against microvesicular steatosis in alcoholic liver disease model (6). Similarly, Zhao et al. showed that STC2 ameliorated hepatosteatosis and hypertriglyceridemia in obese mice through the activation of Signal transducer and activator of transcription 3 (STAT3) signaling pathway (10). STC2 has also been involved in deregulation of glycaemia in obese mice as well as in type 2 diabetes mellitus patients (8). Additionally, it was speculated that STC2 might play role in glucose uptake and metabolism, glycogen storage and triacylglycerol synthesis in both brown and white adipose tissues (44, 63). Taken together STC2 exhibits pro-survival effect in various model systems.

### Tumor Biology

The human *STC2* gene has been mapped to 5q35.1, which is linked with tumor progression and metastasis (31, 37). STC2 expression has been associated with two essential conditions namely hypoxia and ER stress associated with tumor microenvironment (5, 7). Role of STC2 in human cancers has been studied from two different perspectives namely expression of STC2 in specific cancer models and cell lines, and STC2's function in cell growth, differentiation and apoptosis. Several reports suggest elevated expression of STC2 in human hepatocellular carcinoma, neuroblastoma, breast cancer, colorectal cancer, renal cell carcinoma, esophageal squamous cell cancer, and prostate cancer (41, 64–69). Additionally, expression of STC2 was correlated with tumor invasion, metastasis and size in gastric cancers and hepatocellular carcinoma (70). In gastric cancer patients, STC2 expression in circulating tumor cells as well as serum STC2 levels were positively correlated with pathological diagnosis and prognosis (71, 72). Study by Wang et al. indicated elevated levels of STC2 in hepatocellular carcinoma tissues and were related to tumor size and multiplicity of hepatocellular carcinoma (68). Both STC2 mRNA and protein expression were related to tumor size, stage, metastasis, and differentiation in hepatocellular carcinoma. The hepatocellular carcinoma patients with higher expression of STC2 also had shorter median survival time (68). Furthermore, ectopic expression of STC2 promoted hepatocellular carcinoma cell proliferation and colony formation. STC2 expression also regulated G1 to S phase transition and altered protein levels of cyclin D1 and pERK1/2 suggesting direct role of STC2 in hepatocellular carcinoma progression and metastasis (70). In pancreatic cancer, STC2 expression was positively correlated with tumor size and lymph node metastasis and negatively correlated with 5 years survival rate studied in 98 case samples (73). Furthermore, overexpression of STC2 promoted the proliferation, migration, and invasion of pancreatic cancer by inducing epithelial–mesenchymal transition (73). In colorectal cancer, STC2 promoted the epithelial-mesenchymal transition of colorectal cells via AKT-ERK signaling pathway (40). STC2 overexpression was associated to nasopharyngeal carcinoma malignancy and poor prognosis including higher potential of progression and distant metastasis (74). Using CNE2 cell line model as well as tumor samples from 94 patients, it was shown that STC2 promoted post-radiation survival, migration, and invasion of nasopharyngeal carcinoma (74, 75). It was also reported that overexpression of STC2 promotes ovarian cancer growth as well as promotes tumorigenicity and growth in colon cancer (76). On contrary, knockdown of STC2 under hypoxic conditions reverses migration of colon cancer (77). STC2 has also been implicated in promoting head and neck squamous cell carcinoma metastasis via regulation of PI3K/AKT/snail signaling pathways (78). STC2 was also positively correlated with metastasis and progression of lung cancer (42), but surprisingly, a knockdown of STC2 in H460 lung cancer cell line attenuated hydrogen peroxide induced oxidative stress and ROS levels suggesting putative protective role of STC2 in redox regulatory system of lung cancer (42). STC2 also suppressed breast cancer cell migration and invasion by PKC/claudin1 mediated signaling (41, 43). STC2 expression was also associated with positive outcome in male breast cancer (79). Taken together, except in breast cancer, STC2 expression is a potential prognostic marker for variety of cancers which promotes tumor cell growth, invasion and migration.

## Conclusions

**Figure 2:**
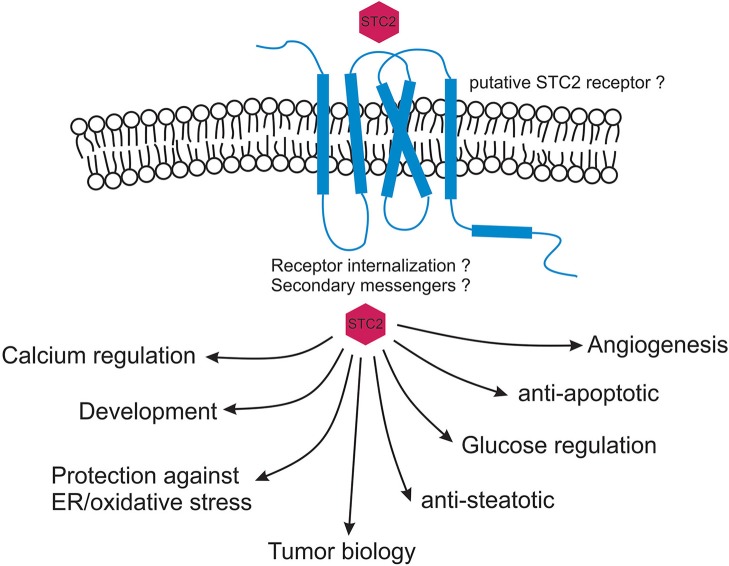
Myriad functions of stanniocalcin 2. STC2 is predicted to function in an autocrine and / or paracrine fashion. STC2 interaction with a putative receptor is speculated based on current immunological data. Once internalized, STC2 plays critical roles in maintaining intracellular calcium and phosphate levels, cytoprotects against ER/oxidative stress induced apoptosis and maintains glucose homeostasis. STC2 is also an important component involved in vascular development, metabolism, animal development as well as in human cancers.

Originally discovered from Corpus of stannous in fish, stanniocalcin paralogs (*STC1* and *2*) have been identified and cloned from human and rodents and are expressed in almost all the mammalian tissues. Phylogenetic examination revealed that both *STC1* and *STC2* are evolved from a common ancestral gene and there is a possibility of identification of additional stanniocalcin-like agonists across eukaryotes. Biologically STC2 has been shown to play role in calcium regulation, ion transport, growth and development, cell protection, metabolism, angiogenesis, and oncology. Located in ER and Golgi apparatus, the STC2 hormone is predicted to exert its biological function through an autocrine and/or paracrine pathway. Direct molecular and cellular studies are warranted to determine STC2's secretary function as well as to detect its putative receptor. Apart from STC2's dimeric nature and presence of histidines predicted to be involved in divalent metal ion binding very little is known about its three-dimensional structure and interactions with other proteins. With STC2's direct correlation with variety of cancers, it is expected that forthcoming studies will help explore the function and regulatory mechanisms of STC2 in tumor progression and metastasis. With STC2's indispensable role in cytoprotection against ER/oxidative stress induced apoptosis it is also imperative to study molecular pathways associated with this protein (Figure 2). Finally, given STC2's involvement in intracellular calcium regulation and protection against steatosis, hypertriglyceridemia, ischemia, and hypertonic stresses its pathophysiological characterization will be helpful in designing clinically relevant therapeutic strategies against plethora of diseases.

## Author Contributions

The author confirms being the sole contributor of this work and has approved it for publication.

### Conflict of Interest

The author declares that the research was conducted in the absence of any commercial or financial relationships that could be construed as a potential conflict of interest.
